# Case report: Presumed contact lens-induced intracorneal hemorrhage in a diabetic dog

**DOI:** 10.3389/fvets.2022.959782

**Published:** 2022-07-22

**Authors:** Sunjun Jung, Shin Ae Park

**Affiliations:** ^1^Department of Veterinary Surgery, College of Veterinary Medicine, Jeonbuk National University, Iksan, South Korea; ^2^Department of Veterinary Clinical Sciences, College of Veterinary Medicine, Purdue University, West Lafayette, IN, United States

**Keywords:** bandage contact lens, diabetes mellitus, indolent ulcer, intracorneal hemorrhage, SCCED

## Abstract

A 10-year-old castrated male miniature poodle dog with diabetes mellitus was presented for a week history of blepharospasm and epiphora in the right eye. The spontaneous chronic corneal epithelial defect (SCCED) was diagnosed, and a bandage contact lens was applied following corneal debridement with sterile cotton-tip applicators. In 1 week, SCCED was improving uneventfully, though an annular pattern of intracorneal hemorrhage was observed. The contact lens was removed and the intracorneal hemorrhage resorbed in 4 weeks. To the author's knowledge, this is the first report of presumed contact lens-induced intracorneal hemorrhage characterized by an annular pattern in a dog.

## Introduction

Contact lenses have been used in veterinary patients with corneal diseases to aid in the treatment outcomes of the spontaneous chronic corneal epithelial defect (SCCED) and other types of ulcerative keratitis (1). The lenses protect the cornea from irritation due to anatomic abnormality of eyelids and eyelashes (2). They are also used as a part of post-operative management of symblepharon surgery (3). While contact lens is widely used in veterinary ophthalmology, there is little available veterinary literature pertaining to the side effects of such usage. In human medicine, the literature reports many complications associated with wearing contact lenses such as mechanical trauma, corneal edema, corneal wrinkling, limbal hyperemia, reduced corneal sensitivity, corneal neovascularization, and intracorneal hemorrhage (ICH) (4, 5).

There have been a few reports of ICH in dogs that were suspected to be related to other ocular or systemic diseases including keratoconjunctivitis sicca (KCS), corneal ulcer, systemic hypertension, diabetes mellitus (DM), hypothyroidism, hyperadrenocorticism, coagulation disorder, sepsis, and neoplasia (6, 7). To the best of our knowledge, however, ICH associated with contact lens wear has not been reported in veterinary medicine. The purpose of this case report is to describe an ICH presumed to be related to a bandage contact lens for SCCED treatment in a diabetic dog.

## Case description

**Figure 1:**
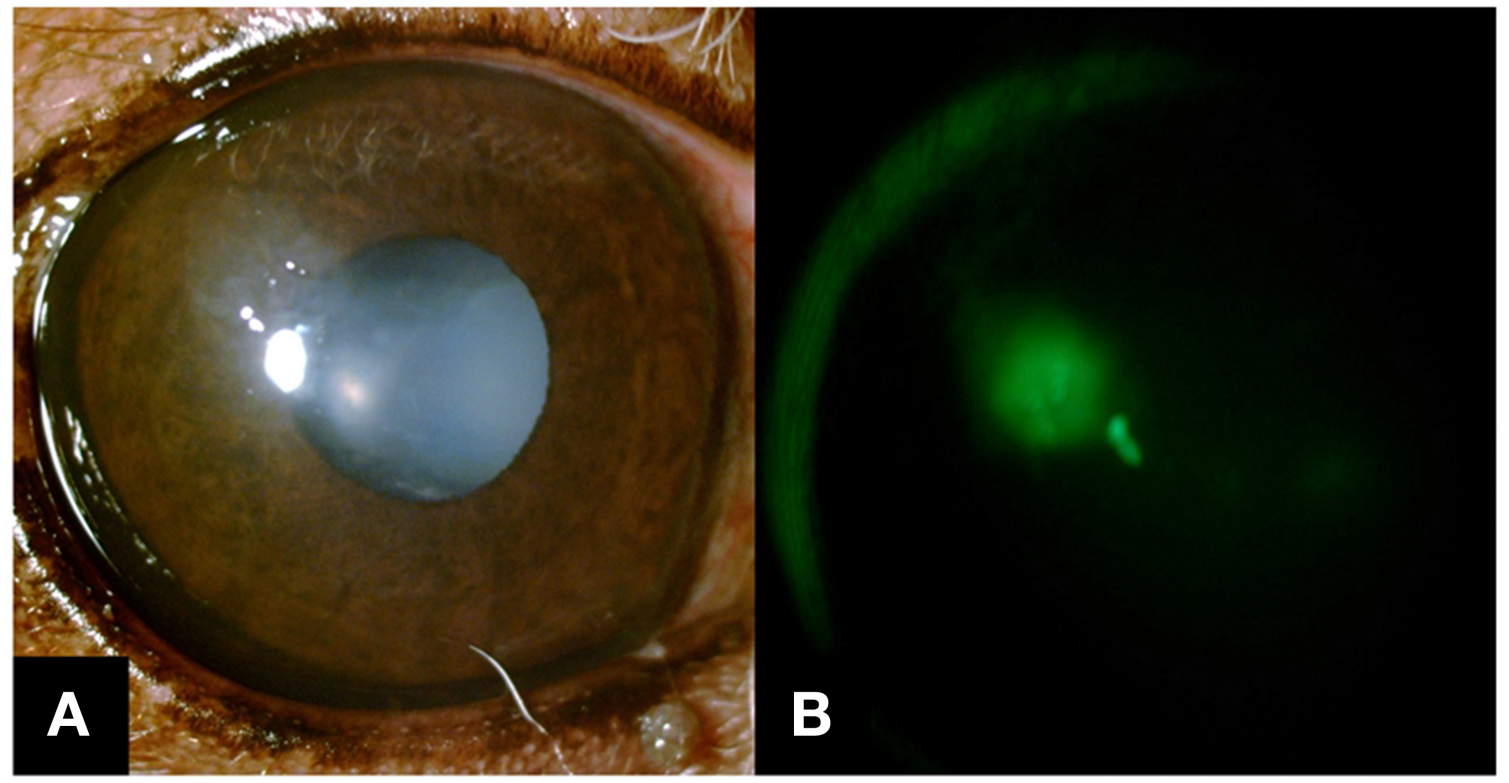
Slit-lamp biomicroscope images were obtained on the day of referral. **(A)** Corneal epithelial defect surrounded by nonadherent epithelium was observed as OD. Not prominent in this image because of the background iris color, but there were a few 0.5 mm superficial corneal vessels along the superior limbus. **(B)** An indistinct margin of fluorescein retention was noted under the nonadherent epithelial lip.

A 10-year-old castrated male miniature poodle dog was referred with a week history of blepharospasm and epiphora in the right eye. Approximately 2 weeks prior to the referral, the dog was diagnosed with DM and was treated with insulin 0.4 IU/kg, BID (Humulin N^®^, Lily Korea, South Korea). On the day of the referral blood, glucose concentration was 260 mg/dl on average (nadir 160 mg/dl) with glycosuria persisted. Neuro-ophthalmic examinations including palpebral reflex, menace response, corneal reflex, dazzle reflex, and direct and indirect pupillary light reflex were normal OU. Schirmer tear test 1 (STT-1) results were 17 mm/min OD and 13 mm/min OS. Intraocular pressure (IOP) measured with a rebound tonometer (Tonovet^®^, iCare USA, Raleigh, NC) was 5 mmHg OD and 10 mmHg OS. Slit-lamp examination OD revealed conjunctival hyperemia, several 0.5 mm of superficial corneal vessels along the superior limbus, an epithelial defect with surrounding non-adherent corneal epithelium in the paraxial cornea, nuclear sclerosis, and incipient cataract. Fluorescein stain was retained underneath the nonadherent epithelium (Figure 1). The surface of the other part of the cornea was smooth and there was no fluorescein stain uptake on the corneal or conjunctiva other than the area of the corneal ulcer. Eyelids, meibomian glands and eyelashes appeared normal. There was no follicle or abnormal irregularity of the palpebral conjunctiva. There was no clinical evidence of infection. Slit-lamp examination OS revealed conjunctival hyperemia, nuclear sclerosis, and incipient cataract. Fluorescein stain was not retained OS. Fundic examination OU was unremarkable.

The spontaneous chronic corneal epithelial defect was diagnosed as OD and the ulcer was debrided with sterile cotton-tip applicators after instillation of topical anesthetic solution (Alcaine^®^, Alcon, Belgium). The loose epithelium was removed from the center of the erosion to the outward. At the end of the procedure, a bandage contact lens (Acuvue^®^ Oasys™ with Hydraclear™ Plus, Johnson and Johnson Vision Care Inc, Jacksonville, FL) was applied (Base-Curve: 8.5 mm, Diameter: 14.3mm, Dk/t: 121, Power: −0.5 D). The tightness of the contact lens was evaluated *via* a push-up test and regarded as suitable for the patient. To prevent infection and lubricate the cornea, topical ofloxacin (Ocuflox^®^, Samil, South Korea) one drop, q8h OD and topical artificial tear (Hyalein^®^, Santen, Japan) one drop, q8h OU were prescribed for 7 days. An Elizabethan collar was placed until the end of the treatment.

**Figure 2:**
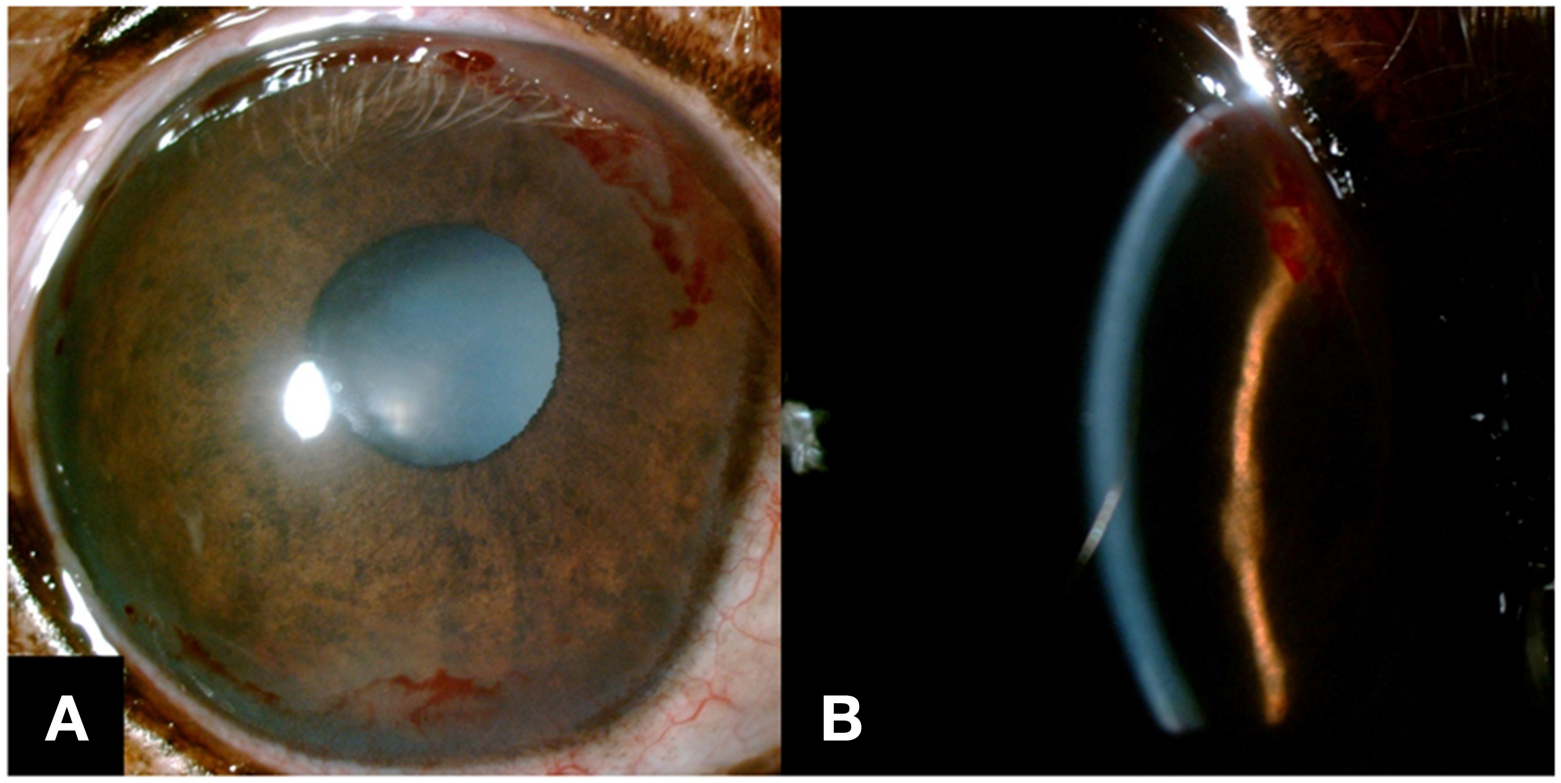
Slit-lamp biomicroscope images were obtained a week after the bandage contact lens wear. **(A)** ICH in an annular pattern was noted as OD. **(B)** Subepithelial hemorrhage was noticed on the slit beam image.

After a week, no more blepharospasm or ocular discharge was noticed OD. Before the ocular examination and after instilling a drop of topical anesthetic solution, the bandage contact lens was removed. IOP was 6 mmHg OD and 10 mmHg OS. Slit-lamp examination of OD revealed mild corneal edema at the previous ulcer occurred site but the nonadherent corneal epithelial margin was absent. Additionally, subepithelial ICH, which had not been detected at the previous visit, was observed in an annular pattern involving ~270° of the peripheral cornea (Figure 2). The fluorescein stain result was negative. Clinical signs of DM including polyuria and polydipsia were resolved, and the result of the blood glucose curve showed a controlled level of blood glucose concentration (average 232 mg/dl, nadir 128 mg/dl). No more glycosuria was detected. Based on the results of complete physical examination and diagnostics including blood pressure, systemic bloodwork including complete blood cell count, blood gas analysis, serum chemistry, PT, and aPTT, adrenocorticotropic hormone stimulation test, and thyroid test (T4, fT4, TSH) that was all within normal range, we were able to rule out the following systemic diseases: hyperadrenocorticism, hypothyroidism, systemic hypertension, or coagulopathy, which could potentially be related to corneal hemorrhage in dogs other than DM (6). The patient was continued with topical ofloxacin and artificial tears for 7 more days.

**Figure 3:**
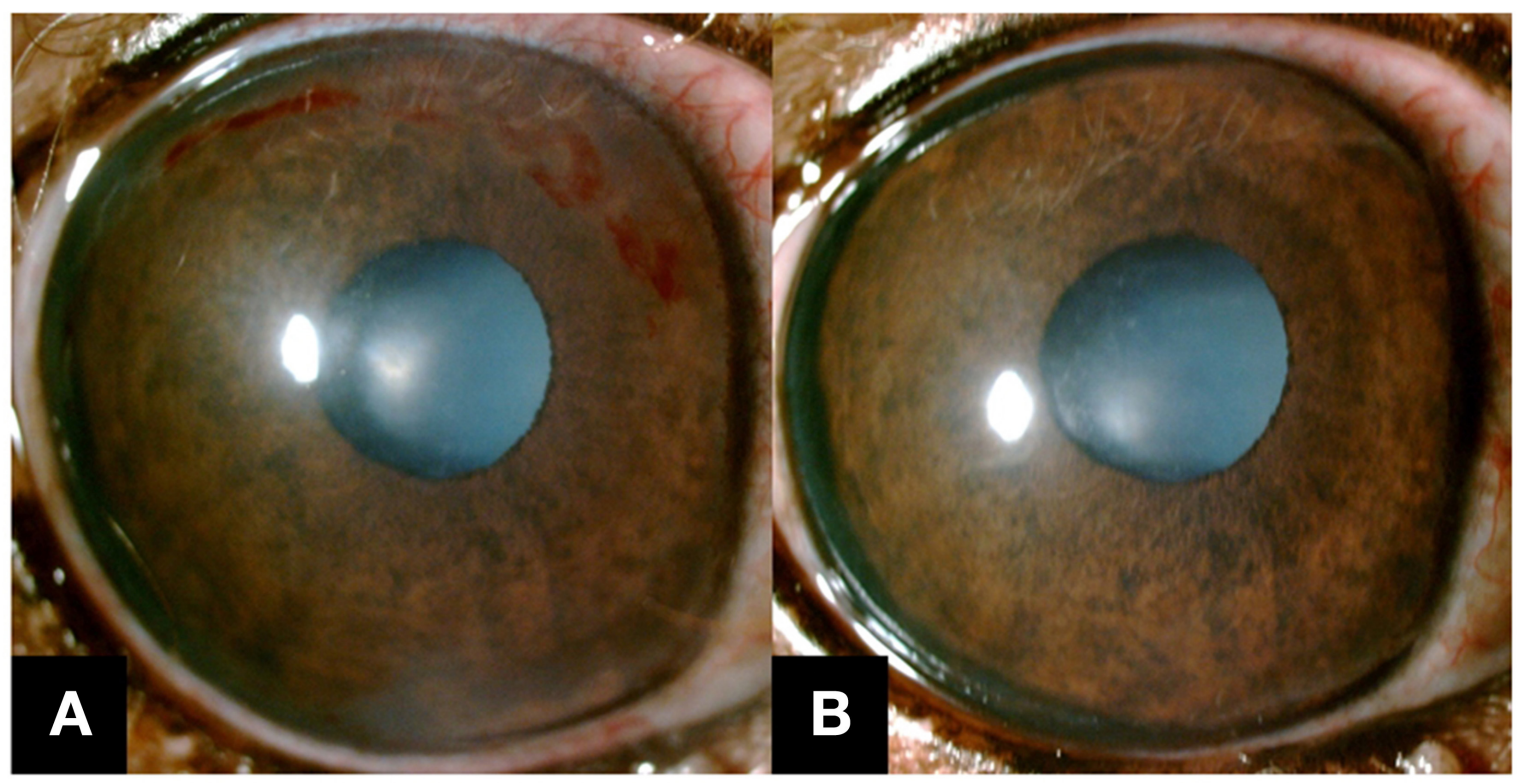
Slit-lamp biomicroscope images were obtained after the removal of the contact lens. **(A)** After a week, ICH was still present in the superior cornea, but the rest was resolved. **(B)** After a month, complete resorption of ICH was observed, and the epithelium of the cornea appeared stable.

In a follow-up examination in a week, the owner reported that the dog's DM was well-managed and there was no evidence of ocular discomfort. IOP was 12 mmHg OU. On slit-lamp examination OD, the epithelial surface of the cornea was stable, and corneal edema was almost resolved, but mild fibrosis was observed. ICH was resorbed but still observed from the 11 to 3 o'clock position of the cornea (Figure 3A). Topical ofloxacin was discontinued but considering the possible tear film instability associated with DM, topical artificial tear at one drop, q8h OU was continued.

One month later, a complete resolution of ICH was observed in a slit lamp examination (Figure 3B). IOP was 13 mmHg OU. STT-1 result was 23 mm/min OD and 25 mm/min OS. While a subtle progression of cataracts was noticed in OU, it was still in the incipient stage.

Informed consent to perform all the examinations and treatments including contact lens placement was obtained from the owner of the animal.

## Discussion

To the authors' best knowledge, this is the first report of ICH presumed to be related to bandage contact lens with its characteristic annular pattern in a dog. ICH is occasionally observed in dogs with corneal vascularization. However, those are mostly focal, not perilimbal annular patterns as shown in this report (6, 7). In the present case, corneal neovascularization preceded contact lens use and is suspected to be associated with the healing process of corneal ulcers. In human medicine, though, one of the most common side effects of contact lens wear is corneal vascularization (8, 9). Suggested possible factors of contact lens-induced corneal vascularization are microtrauma from the contact lens, corneal edema, extended wear, and corneal hypoxia (8, 10). There are many reports of corneal neovascularization due to contact lens wear in humans, however, reports of ICH related to contact lenses are relatively uncommon (4, 5, 8).

The annular pattern of ICH in the present case resembles a report of annular pattern subepithelial ICH following contact lens wear in a human patient (4). In this report, corneal pannus was found in all quadrants, which was presumed to be related to annular patterned ICH. ICH reported in dogs is limited to focal areas within 1 or 2 all corneal quadrants with no reports of ICH with an annular pattern (6, 7). In this patient, we suspect that the main causes of ICH are, as suggested in human reports (4, 5), the friction of the contact lens with preexisting corneal vessels and the increased fragility of newly formed vessels. Additionally, ICH would exacerbate by the high susceptibility in diabetic patients to bleeding due to upregulation of macrovascular MMP-9 activity (11). The other possible factor that leads to ICH, in this case, is corneal hypoxia due to contact lens wear. Hypoxia is a well-known cause of angiogenesis, and it is also known to interfere with the integrity of the vessels resulting in hemorrhage (12). It is reported that the elements that are closely related to contact-lens-induced corneal hypoxia are low-gas permeable, thick, and extended-wear of tightly fitting contact lenses (8). Although the contact lens used in this case had high oxygen transmissibility, a hypoxic condition might have been induced on the ocular surface by the wearing of the lens continuously over 7 days (13). DM in this patient may have exacerbated corneal hypoxia as low oxygen consumption in the diabetic cornea occurs in association with the impaired oxygen transport observed in diabetic patients (14). Although the exact pathogenesis of corneal hypoxia in DM still needs to be investigated, it is suggested that the activated sorbitol pathway by high glucose levels leads to an imbalance in the ratio of oxidized to reduced nucleotides (14).

In general, ICH does not require specific treatment and resolves spontaneously with time. The mainstay of ICH treatment in humans is to remove the causative contact lens (4). Although rare, there are reports of humans undergoing surgery for contact lens-related ICH such as corneal epithelial denudation, and lamellar or penetrating keratoplasty (4, 5). In the present case, the ICH was resolved in approximately a month with the discontinuation of the contact lens and the installation of artificial tears. It has been reported that the viscous lubricating eyedrops help suppress corneal vascularization secondary to contact lens wear (15). Concurrent use of viscous tear replacement with contact lens application may help, especially with high-risk patients, prevent contact lens-associated ICH. More prophylactically, other treatment options instead of contact lenses may need to be considered in patients with a high risk of corneal hemorrhage. In the present case, the other therapeutic option for SCCED such as grid keratotomy, anterior stromal puncture, superficial keratectomy, or thermal cautery could be applied instead of a bandage contact lens (1).

In summary, this is the first report presenting contact lens usage as a potential contributing factor to ICH development in a dog. While most ICH in dogs resolves spontaneously without complications, it is recommended that patients receiving contact lens treatment have thorough follow-up examinations of the ocular surface.

## Data Availability Statement

The original contributions presented in the study are included in the article/supplementary material, further inquiries can be directed to the corresponding author.

## Author contributions

SJ contributed to case management and manuscript preparation. SJ and SP contributed to the case analysis, data curation, and manuscript editing. All authors contributed to the article and approved the submitted version.

## Funding

This study was partially funded by the National Eye Institute/National Institutes of Health grant K08EY030950.

## Conflict of interest

The authors declare that the research was conducted in the absence of any commercial or financial relationships that could be construed as a potential conflict of interest.

## Publisher's note

All claims expressed in this article are solely those of the authors and do not necessarily represent those of their affiliated organizations, or those of the publisher, the editors and the reviewers. Any product that may be evaluated in this article, or claim that may be made by its manufacturer, is not guaranteed or endorsed by the publisher.
